# Improving Parent–Child Relationships for Young Parents in the Shadow of Complex Trauma: A Single-Case Experimental Design Series

**DOI:** 10.1007/s10578-022-01379-8

**Published:** 2022-06-27

**Authors:** Jacqueline Kemmis-Riggs, Adam Dickes, Kris Rogers, David Berle, John McAloon

**Affiliations:** 1 Graduate School of Health, University of Technology Sydney, 100 Broadway, Ultimo, NSW 2007 Australia; 2 School of Psychiatry, The University of NSW, Sydney, NSW Australia; 3 School of Population Health, The University of NSW, Sydney, NSW Australia

**Keywords:** Teenage parents, Intervention, Adverse childhood experiences, Parent–child relationships, Complex trauma

## Abstract

This study provides a preliminary evaluation of a dyadic intervention for young parents with a history of complex trauma, *Holding Hands Young Parents* (HHYP). Four mothers (17–22 years) and toddlers (12–33 months) completed the intervention, designed to improve parent–child relationships, parental self-regulation, self-efficacy and mental health, and child behaviour/emotional problems. An A–B single case experimental design series with follow-up and randomised baseline, used observational and self-report measures throughout. Linear mixed models demonstrated improvement in reciprocity and parental sensitivity over the treatment phase, with no evidence of shifts in scores at beginning or end of treatment. There was no evidence for changes in child engagement, negative states, intrusiveness or withdrawal. Reliable Change Index indicated improvement in parent-reported self-regulation, self-efficacy, stress and child emotional/behavioural problems from baseline to follow-up for all four mothers; depression showed reliable change for three. This study demonstrates relational change between young parents and their toddlers and provides preliminary data on the HHYP protocol.

**Supplementary Information:**

The online version contains supplementary material available at 10.1007/s10578-022-01379-8.

## Introduction

Complex trauma, also known as developmental trauma, is defined as the experience of multiple and developmentally adverse traumatic events, most often of an interpersonal nature and with early-life onset [1]. These exposures often occur within the child’s caregiving system and can have extremely negative impacts on child wellbeing in the short and longer term. Children who grow up with abuse, severe neglect and extreme distress are at greater risk of poor physical and mental health [e.g., 2, 3], greater difficulties in parenting behaviours [4–6], and continuity of maltreatment [7–9].

Young parents who have experienced complex trauma, especially adolescent parents, are at particular risk of poor outcomes. Adolescent parents have reduced educational opportunities, unstable housing, socioeconomic disadvantage and limited social support [10–13]. Children of young parents are also at greater risk of poor outcomes, such as low birthweight, increased morbidity in the first year of life, more behaviour problems, higher risk of removal into care, more likely to be born into and continue to live in social and economic disadvantage and more likely to become young parents themselves [13–19]. Thus, it is vital that young parents who have experienced complex trauma are supported to ensure better outcomes for their children and to break intergenerational cycles of trauma and maltreatment.

A wealth of research indicates that safe, stable, and nurturing relationships for infants and children protect them from the negative effects of stress and adversity and can help to break intergenerational cycles of trauma, abuse and neglect [20, 21]. Early socio-emotional development occurs within the context of the parent–child relationship and higher quality parent–child relationships are positively related to children’s socio-emotional development [22–25]. Thus, enhancing parenting behaviours provides children with an improved social environment that supports the development of secure attachment and socio-emotional capacities. One key aspect of parenting behaviour is sensitivity, defined as the capacity to perceive and interpret the meaning behind the child’s signals, and to respond to them promptly and appropriately [26]. It is central for the development of secure attachment and for promoting healthy child socio-emotional development [22–25, 27, 28].

### Holding Hands Young Parents

The Holding Hands Young Parents intervention [HHYP; 29] was developed to support young parents who have experienced complex trauma and their toddlers. HHYP aims to improve the quality of the parent–child relationship, increase young parents’ self-regulation and self-efficacy, and support them in responding more effectively to child behavioural and emotional problems and is founded on principles of attachment theory [26, 30, 31], the biobehavioural synchrony model [32, 33], social learning theory [34, 35] and coercion theory [36]. Thus, it shares commonalities with multiple evidence-based parenting programs [e.g., 37–40]. The intervention also incorporates several adaptations to meet the needs of younger parents who have experienced complex trauma, including an explicit focus on skills to improve parent emotion regulation and tailored education about child socio-emotional development. HHYP includes several delivery methods that have robust empirical support in trials with families at risk of, or with a history of, maltreatment, including video-feedback [e.g., 41–43] and in-vivo coaching [e.g., 44–46].

Systematic reviews of randomised controlled trials evaluating short-term parenting interventions targeted specifically at adolescent parents have shown that these interventions may be effective in improving maternal sensitivity and parent–child interactions [47, 48]. However, reviewers have suggested that they may have a limited role in supporting teenage parents and that increased benefit may result from their use in conjunction with more intensive services that target broader outcomes related to social exclusion [48]. Therefore, this intervention was developed to be delivered within the context of multifaceted service responses that provide ongoing case management, home-visiting and support for housing, educational, vocational and/or life skills needs.

We aimed to examine the effects of HHYP on parent–child relationship quality, parent self-regulation and self-efficacy, parent mental health, and child behaviour problems for young parents who have experienced complex trauma and their toddlers. Considering the early stage of intervention development and the vulnerable and heterogeneous nature of the population, a series of A–B comparisons plus follow-up with randomised baseline periods was deemed the most appropriate. Given the evidence-base for the components in the intervention, it was expected that indices of parent–child relationship quality would improve. Specifically, we expected that parent sensitivity, child engagement and dyadic reciprocity would increase and parental intrusiveness, child withdrawal and dyadic negative states would decrease over the course of the intervention. We also expected the intervention would yield positive effects on parent self-regulation and self-efficacy, parent mental health, parenting stress, and child behaviour problems (i.e., child internalising and externalising problems).

## Method

### Participants

Following approval from the University of Technology Sydney Human Research Ethics Committee (ETH18-2949), participants were recruited from a community young parents’ program that provides multifaceted services, including case management, home visiting, housing support, parent education and parenting playgroups (Program name has been withheld to protect participant confidentiality). Recruitment commenced in July 2018. The HHYP intervention was delivered from August 2018 until October 2019. Treatment was conducted at the Family Child Behaviour Clinic at the University of Technology Sydney, Australia. No compensation was provided to participants, however, they participated in the clinical intervention at no cost. Participants were eligible for the study if they met the following inclusion criteria (a) parent was aged between 16 and 25 years with a child aged between 6 and 48 months; (b) parent reported difficulties in their relationship with their child; (c) parent was assessed as having a history of complex trauma. Complex trauma was defined as having a history of child maltreatment, exposure to domestic violence or drug and alcohol abuse within family environment. This was initially screened using information provided by community program staff and substantiated by self-report during the assessment stage, using the Child Trauma Questionnaire [49] and clinical interviews. Participants were ineligible for inclusion in the study if they met any of the following criteria (a) child had a prior diagnosis of Severe Intellectual Disability, Autism Spectrum Disorder (level 2 or 3), deafness or blindness; (b) parent had current untreated substance abuse or dependence; (c) parent had suicidal or homicidal ideation or significant risk of harm to self or others; or (d) parent had current psychotic symptoms or psychotic disorder.

The final sample included 4 mother-toddler dyads. Eight dyads were referred to the intervention by staff at the community program to improve their attachment relationships, self-regulation, reflective capacity, and challenging toddler behaviours and screened for eligibility by the lead researcher (JKR). Eligible dyads were then scheduled for an assessment. Parents provided informed consent for themselves and their children at their first visit. Of the 8 dyads referred, all were eligible and completed the baseline assessment. Four discontinued in the assessment stage or in the first few weeks of the intervention due to environmental factors.^1^ The demographic characteristics of the final sample are as follows. Mothers’ ages ranged from 17 to 22 years and their toddlers were aged between 13 and 33 months. All mothers were teenagers when they gave birth. They all spoke English as their main language at home. All mothers and their toddlers were born in Australia. Two described their ethnic background as Aboriginal Australian and two described their background as Caucasian. Mothers’ highest education ranged from Year 6 to Year 10. Table 1 provides additional information about mothers’ history of childhood trauma assessed at baseline.

**Table 1:** Means and standard deviations on the Childhood Trauma Questionnaire (CTQ-SF) assessed at baseline

CTQ-SF subscale	Mean	SD	Clinical range
Emotional abuse	14.50	7.14	Severe
Physical abuse	14.50	6.18	Severe
Sexual abuse	12.75	7.23	Severe
Emotional neglect	10.50	2.87	Moderate
Physical neglect	10.25	3.70	Severe

### Study Design and Procedure

**Fig. 1:**
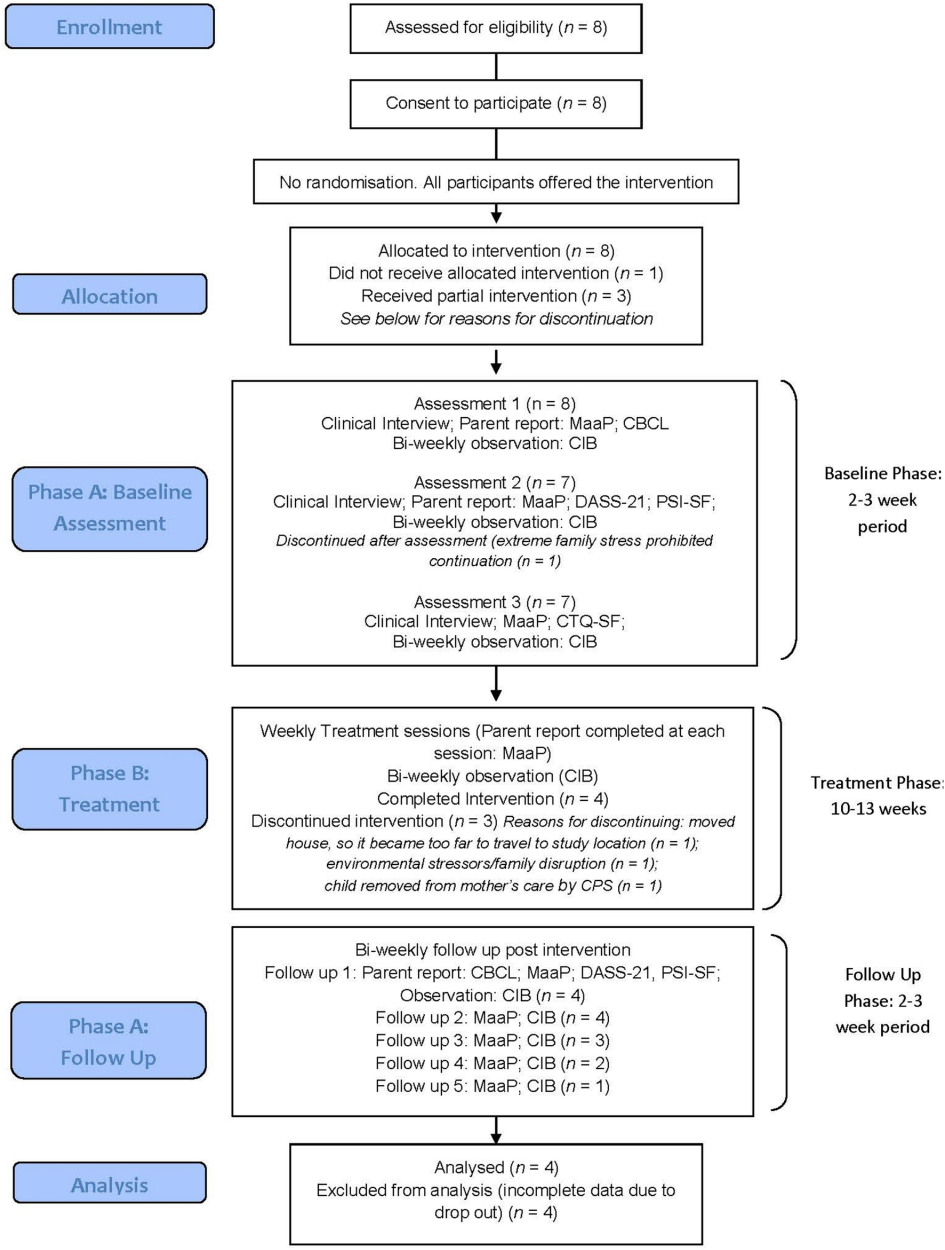
Consort flow diagram of intervention phases and measures collected throughout the study. *CBCL* Child Behaviour Checklist; *CIB* Coding Interactive Behaviour scale; *CTQ-SF* Child Trauma Questionnaire; *DASS-21* Depression, Anxiety Stress scale; *MaaP* Me as a Parent scale; *PSI-SF* Parenting Stress Index 4th Edition Short Form

The study design involved a series of A–B comparisons plus follow-up with randomised baseline periods, replicated across four dyads. The recruitment of a vulnerable and hard-to-reach population and limitations in intake in the service that we utilised to identify prospective participants meant it was difficult to engage more than two dyads concurrently. We report findings using the single-case reporting guidelines in behavioural interventions [SCRIBE; 50]. Participants were randomly assigned to baseline phases lasting 2 or 3 weeks. Parents participated in repeated biweekly observational assessments and completed weekly parent-report questionnaires via a secure, online survey platform during each study phase. During the treatment phase, parents completed all measures at the start of each session. Figure 1 shows the consort flow diagram.

### Measures

Parents participated in a semi-structured clinical interview assessing presenting concerns, child and family details, child health and development, family relationships, social support, and parents’ family history and early childhood environment. Parents also completed a range of structured questionnaires and participated in observational assessments at baseline, throughout treatment and at follow-up.

#### Demographics

Demographics included parent and child age, gender, ethnic background, educational background, language spoken at home and experience of out of home care.

#### Parent Trauma History

The Childhood Trauma Questionnaire, short form [CTQ-SF; 49] is a 28-item screening for maltreatment histories, the presence and severity of emotional, physical and sexual abuse, and emotional and physical neglect. The CTQ-SF has been validated for adults and adolescents, demonstrating strong psychometric properties in clinical and non-clinical populations [49, 51, 52]. In a community sample, Cronbach’s alphas were: 0.87 for Emotional abuse, 0.83 for Physical abuse, 0.92 for Sexual abuse, 0.91 for Emotional neglect, and 0.61 for Physical neglect [49]. The CTQ manual provides thresholds for four levels of abuse/neglect for each subscale: None; Moderate; Severe; Extreme.

#### Parent–Child Relationship

The Coding Interactive Behaviour [CIB; 53] is a global observational rating system for social behaviour and was the primary measure for this study. It includes 45 codes rated from 1 to 5, organised into several composites that index important relationship aspects, including parental sensitivity, parental intrusiveness, child engagement, child withdrawal and dyadic interactions of reciprocity and negative states. The CIB is typically applied to free social interactions between two partners. The CIB has been validated in multiple studies across numerous cultures with children ranging in age from newborn to adolescents, demonstrating good psychometric properties [54]. Five minutes of free-play between parent and child were video-recorded at the beginning of each session. The videos were coded after treatment was completed. Three coders who had completed certification in CIB coding and trained to 85% reliability on all codes coded the interactions. Two coders were independent to this study and blind to mother and child status and session order. The third coder (JKR) was the primary author and clinician delivering the intervention. Inter-rater reliability was computed for over 39% of the interactions and inter-rater agreement was > 85% on all codes (intra-class *r* = 0.90, range between 0.80 and 0.98). Composites, codes included in each composite, and internal consistency for the current sample were as follows: *Parent intrusiveness* includes forcing, overriding, anxiety, and parent-led interactions (α = 0.73). *Parent sensitivity* includes acknowledging, elaborating, parent gaze/joint attention, positive affect, vocal appropriateness, appropriate range of affect, resourcefulness, praising, affectionate touch, and parent supportive presence (α = 0.85). *Child social engagement* includes child gaze/joint attention, child positive affect, child affection to parent, alert, fatigue (reversed), child vocalization, child initiation, competent use of the environment, and creative symbolic play (α = 0.79). *Child withdrawal* includes negative emotionality, withdrawal, emotion lability, child avoidance of parent (α = 0.64). *Dyadic reciprocity* includes dyadic reciprocity, adaptation-regulation and fluency (α = 0.96). *Dyadic negative states* includes constriction and tension (α = 0.62).

#### Parent Self-regulation

The Me as a Parent Scale [MaaP; 55] is a 16-item self-report questionnaire comprising 4 subscales measuring global beliefs about self-efficacy, personal agency, self-management, and self-sufficiency, theorised to constitute parent self-regulation perceptions. Scores are calculated for an overall total and each subscale. Parents rated items on a 5-point Likert-type scale from 1 (*strongly disagree*) to 5 (*strongly agree*). Items were averaged so that higher scores indicated higher parenting self-regulation. Questions ask parents how strongly they agree or disagree with statements such as “I know I am doing a good job as a parent” and “I meet my expectations for providing emotional support for my child”. The MaaP has been validated for use in the Australian context, demonstrating good psychometric properties (Hamilton et al. 2014). In the present study, Cronbach’s alpha were: Self-Efficacy; α = 0.84; Personal Agency, α = 0.77; Self-Management, α = 0.84, Self-Sufficiency, α = 0.91; and Total, α = 0.94. The original study protocol (available from the primary author) had also planned to use 4 questions from this scale to be administered daily using a phone app, during each phase. However, feedback from all participants demonstrated this was not acceptable and too burdensome given their other life stressors.

#### Mental Health

The Depression Anxiety and Stress Scale [DASS-21; 56] is a 21-item scale is comprised of three subscales relating to Depression (symptoms associated with dysphoric mood such as anhedonia, hopelessness, and low self-esteem), Anxiety (symptoms relating to anxious affect, such as shakiness), and Stress (general tension, irritability). Higher score are indicative of greater psychopathology, with clinical cut-off scores for each subscale indicative of mild, moderate, severe, and extremely severe symptoms. The scales of the DASS-21 have been shown to have strong psychometric properties and measure both current state and change over course of treatment [57, 58]. The internal consistencies used to calculate the Reliable Change Index (RCI) were estimated using Cronbach’s alpha: α was 0.88 for the Depression scale, 0.82 for the Anxiety scale and 0.90 for the Stress scale [59].

#### Parent Stress

The Parenting Stress Index 4th Edition Short Form [PSI-SF; 60] is a brief version of the Parenting Stress Index [61], a widely used and well-researched self-report measure of parenting stress. The PSI-SF has 36 items from the original 120-item PSI. The PSI-SF yields a Total score and scores on the following subscales: (1) Parental Distress, (2) Parent–Child Dysfunctional Interaction, and (3) Difficult Child. Items are identical to those in the original version. Higher scores on the PSI-SF are indicative of greater dysfunction, with Total Stress scores in the 90th percentile or above representing clinically significant parenting stress [60]. The PSI-SF has demonstrated strong psychometric properties in prior research [62–64]. Cronbach’s alpha were: α = 0.88 for Parental Distress; 0.88 for Parent–Child Dysfunctional Interaction; 0.89 for Difficult Child; and 0.95 for Total Stress [63].

#### Child Emotional and Behavioural Problems

The Child Behaviour Checklist 1.5–5 [CBCL/1.5–5; 65] is a widely used, standardized parent-report measure of child emotional and behavioural problems. The CBCL/1.5–5 consists of 99 items. It produces two broadband scales (externalizing problems and internalizing problems) and a total problems scale. The CBCL has demonstrated strong psychometric properties in prior research with a diverse range of populations [66]. Responders rate items on a scale of 0 (*not true*) to 2 (*very true*). Higher scores indicate higher problems, with scores in the 97th percentile or above represent clinically significant problems [65]. Cronbach’s alpha were: α = 0.89 Internalising scale; 0.92 Externalising scale, 0.95 Total scale [65].

### Treatment

The treatment manual for HHYP [29] incorporates 8 individual 90-min modules that can be delivered in varying order depending on the needs of the family. Each session involved 50 min with the parent and 30 min with the parent and child, during which the therapist coached the parent in free-play with their child from an observation room. This provides an opportunity for parents to practice and strengthen skills learned in session while receiving real-time feedback on skill development. The final 10 min is spent with the parent, child and case manager supporting the young parent. An optional bi-weekly telehealth check-in provides for additional observation of parent–child play and the problem-solving of any pressing parental concerns. The manual includes treatment protocols and handouts for each session. It was expected that dyads would participate in at least 8, once weekly treatment sessions, and that treatment would be paced according to individual need. The number of face-to-face sessions delivered to participants in this study ranged from 10 to 13, and the modules were delivered in the order presented in the manual. Dyad 1 also participated in regular bi-weekly sessions via telehealth, whereas the other dyads engaged in weekly sessions and chose fewer bi-weekly check-ins.

Assessment and treatment was provided by two of the authors (JKR and AD) who are registered psychologists with a Master’s Degree in clinical psychology and experience and training in trauma-informed practice. Authors (JKR, AD, JM) co-developed the treatment. Clinical supervision was provided throughout the study by a senior clinical psychologist (JM) with clinical and operational experience in traumA–Based services for individuals presenting with childhood adversity. The two researchers who served as therapists used an implementation checklist contained in the treatment manual that outlines the core components of the intervention to deliver in each module. All sessions were audio or video recorded, and 25% of the treatment sessions were selected and checked for therapist competence (e.g., rapport, therapist knowledge of content, session management) and inclusion of the manual content for each module. Overall, average competence ratings were high (4.75 on a 5-point scale) and all of the rated sessions included all core content outlined in the HHYP manual. This evaluation of therapist competence was used as an indicator of treatment fidelity [67].

### Data Analysis

A linear mixed model for each of the CIB subscales was estimated separately. The model took the form:

$$Y_{ij} = \beta_{0} + \beta_{1} Session + \beta_{2} Treatment + \beta_{3} Followup + \beta_{4} Session \cdot Treatment + \beta_{5} Session \cdot Followup + u_{i} + e_{ij}$$

where *i* indexes the dyad, j indexes the session number, β_0_ is the estimate of sub-scale score at the first session, β_0_ is the change in score per session in the baseline period, β_0_ is the shift at the beginning of treatment, β_0_ is the shift at the end of treatment, β_4_ and β_5_ are estimates of the additional change over the β_1_ in the treatment and follow-up periods respectively, *u*_*i*_ is the random intercept estimated for each dyad (i.e. the difference in score at session 1 compared to the average), and *e*_*ij*_ is the dyad error term. This model allows the assessment of whether change over time (session) differs between study phases, and whether there is a distinct change in the score at the beginning and end of the treatment period. The 95% confidence interval (CI) is estimated for each of the coefficients. The random intercept and the fixed coefficients were combined to create an estimate for each session number for each dyad. Profile confidence intervals were estimated for coefficients, with no autocorrelation included in the model, and the residual variance modelled as homogenous.

The Reliable Change Index [RCI; 68] was also calculated to evaluate whether there were improvements from baseline to follow-up for each individual case on observed parent–child interaction quality indices (i.e., CIB subscales) and on parent-reported measures of their own mental health symptoms (DASS-21), self-regulation (MaaP), child internalising, externalising and total behaviour problems (CBCL) and parenting stress (PSI-SF). The RCI indicates whether the difference between baseline and follow-up scores is greater than a difference that could have occurred due to random measurement error alone [68, 69]. The formula for reliable change is calculated by dividing participants’ difference scores (pre and post intervention) by the standard error of the measure.^2^ [68]. Values greater than 1.96 represent reliable change. Because there were repeated measures at baseline and follow-up for the CIB and MaaP, the mean scores for each phase were calculated and used to determine the RCI.

## Results

We had planned for 5 follow-up sessions to allow for a longer follow-up period to meet recommendations for robust methodology [i.e., 70]. However, for three dyads, this was not possible. Mother 2 moved out of her supported housing at the end of the intervention and no longer had the support of her caseworker to transport her to the final follow-up sessions. Mother 3 was experiencing substantial family stress and involved with child protection services during the intervention, which interrupted her capacity to engage in the final planned follow-up sessions. Mother 4 was also unable to complete the final follow-up due to family stress and disruption. Nil adverse events occurred during the current study. Table S1 (see supplementary material) indicates the number of sessions for each dyad.

### Parent–Child Relationship

**Fig. 2:**
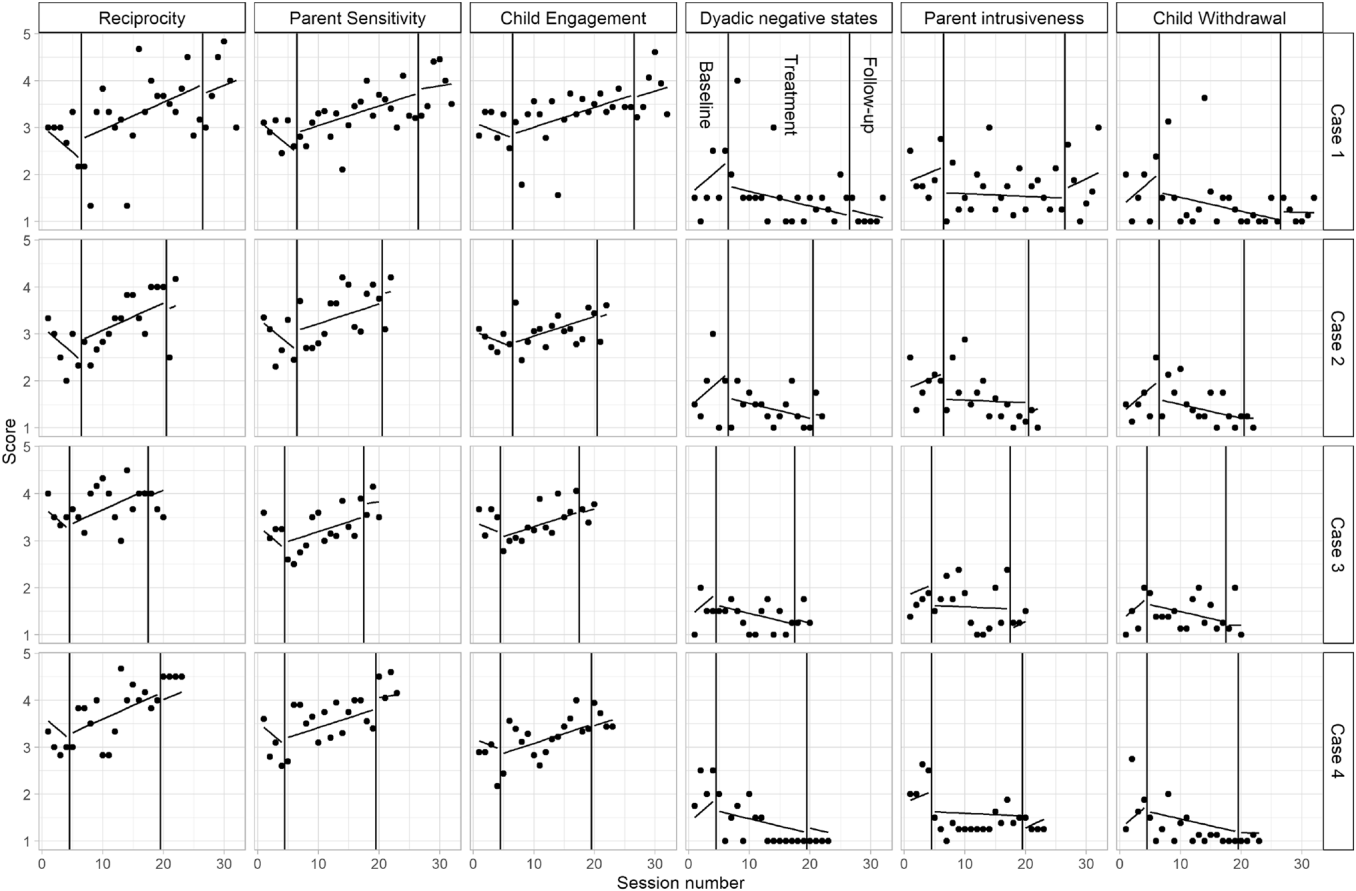
Observed scores (black dots) of CIB scores for all cases over the course of the study (session number) with predicted score for each case at each time-point (black line) from the mixed linear regression model

Table 2 shows the beta coefficients that represent change over sessions, and shifts in the CIB subscales at the beginning and end of treatment, with the observed scores and estimated scores from the linear mixed model in Fig. 2. RCIs for individual cases are reported in Table 3.

**Table 2:** Beta coefficients (fixed effects, with 95% CI) from linear mixed models for the six CIB subscales describing overall change in each study period (change in score per session) and shift at the beginning of each study phase (baseline → treatment, treatment → follow-up)

Coefficient	Reciprocity	Parent sensitivity	Child engagement	Dyadic negative states	Parent intrusiveness	Child withdrawal
ß0 Start score	3.40 (2.74 to 4.05)	3.33 (2.90 to 3.76)	3.20 (2.78 to 3.61)	1.44 (0.97 to 1.91)	1.82 (1.38 to 2.26)	1.29 (0.85 to 1.73)
ß1 Trend during baseline	− 0.11 (− 0.27 to 0.05)	− 0.11 (− 0.22 to 0.01)	− 0.05 (− 0.16 to 0.06)	0.11 (− 0.02 to 0.25)	0.05 (− 0.07 to 0.18)	0.11 (− 0.01 to 0.24)
ß2 Shift at start of treatment	− 0.65 (− 1.32 to 0.02)	− 0.53 (− 1.02 to − 0.04)	− 0.53 (− 1.00 to − 0.06)	0.40 (− 0.17 to 0.97)	− 0.17 (− 0.71 to 0.38)	0.50 (− 0.04 to 1.04)
ß3 Shift at start of follow-up	− 0.71 (− 2.41 to 0.99)	0.11 (− 1.13 to 1.35)	− 0.50 (− 1.70 to 0.69)	0.48 (− 0.94 to 1.90)	− 1.80 (− 3.11 to − 0.49)	− 0.04 (− 1.35 to 1.28)
ß4 Additional trend during treatment	0.17 (0.01 to 0.33)	0.15 (0.03 to 0.26)	0.10 (− 0.02 to 0.21)	− 0.14 (− 0.28 to − 0.01)	− 0.06 (− 0.19 to 0.07)	− 0.14 (− 0.27 to − 0.01)
ß5 Additional trend during follow-up	0.16 (− 0.00 to 0.33)	0.13 (0.00 to 0.25)	0.09 (− 0.03 to 0.21)	− 0.14 (− 0.28 to 0.00)	0.01 (− 0.13 to 0.15)	− 0.11 (− 0.25 to 0.02)

**Table 3:** Reliable Change Indices (RCI) for observed and parent-report outcome measures

Variable	Case 1	Case 2	Case 3	Case 4
CIB reciprocity	− 8.58*	− 4.89*	− 1.05	− 10.98*
CIB parent sensitivity	− 5.28*	− 3.90*	− 2.17*	− 6.41*
CIB child engagement	− 3.54*	− 1.50	− 0.50	− 3.71*
CIB Dyad negative states	1.33	0.59	0.16	2.44*
CIB parent intrusiveness	0.82	2.62*	1.09	3.30*
CIB child withdrawal	2.01*	2.10*	0.13	3.71*
MaaP total	− 7.28*	− 5.70*	− 5.70*	− 7.12*
MaaP—Self-Sufficiency	− 3.58*	− 2.68*	− 2.68*	− 6.25*
MaaP—Self-Efficacy	− 2.73*	− 2.73*	− 2.12*	− 4.56*
MaaP—Personal Agency	− 2.66*	− 0.66	− 3.76*	− 2.77*
MaaP—Self-Management	− 5.26*	− 5.58*	− 1.86	− 1.09
DASS—Depression	2.11*	3.16*	4.22*	1.05
DASS—Anxiety	1.13	4.52*	1.13	− 3.39*
DASS—Stress	6.39*	6.39*	3.19*	7.45*
PSI—Total	3.84*	6.43*	1.24	1.86
PSI—Parent Distress	2.68*	2.46*	1.34	0.89
PSI—CDI	0.46	2.06*	0.92	0.92
PSI—Difficult Child	3.74*	0.44	0.00	1.54
CBCL Total	6.39*	6.39*	7.99*	10.22*
CBCL Internalising	1.28	1.92	0.64	1.07
CBCL Externalising	2.27*	0.25	3.79*	4.04*

*CBCL* Child Behaviour Checklist [65]; *CIB* Coding Interactive Behaviour scale [53]; *DASS* Depression Anxiety and Stress Scale [56]; *MaaP* Me as a Parent Scale [55]; *PSI-SF* Parenting Stress Index 4th Edition Short Form [60]

Bold* represents reliable change from baseline to follow-up

#### Dyadic Reciprocity

There were evidence of change in reciprocity in the treatment phase relative to the baseline phase [difference of 0.17 (95% CI 0.01–0.33) per session], and marginal evidence of a similar change during the follow-up period [0.16, (95% CI − 0.001 to 0.33], and no evidence of shifts at the beginning and end of treatment.

#### Parent Sensitivity

Parental sensitivity improved in the treatment phase (0.15/session, 95% CI 0.01–0.33) and follow-up (0.16, 95% CI 0.003–0.25), but there was no evidence of a shift at the beginning and end of treatment.

#### Child Engagement

There was no evidence across the cases of a difference over sessions in child engagement subscale, or of a shift at the beginning and end of treatment.

#### Dyadic Negative States

Overall there was no evidence of changes in dyadic negative states in any of the study phases, or of shifts at the beginning and end of treatment.

#### Parent Intrusiveness

Overall there was no evidence of changes in intrusiveness states in any of the study phases, or of shifts at the beginning and end of treatment.

#### Child Withdrawal

Overall there was no evidence of difference in change in child withdrawal score over sessions in any of the study phases, or of a shift at the beginning or end of treatment phase.

### Parent Self-report Outcomes

Table 3 shows RCI results for the parent-reported outcomes. As expected, all four participants demonstrated reliable change from baseline to follow-up on the MaaP Total scale, the Self-Sufficiency and Self-Efficacy subscales. Three showed improvements on the Personal Agency subscale and two showed improvement on the Personal Management subscale.

As expected, on the DASS-21, all four parents demonstrated reliable change on Stress. Three showed improvement on Depression and one showed improvement on Anxiety. One parent (P4) reported an increase in anxiety. Qualitative feedback indicated this related to additional external factors impacting her anxiety levels towards the close of the intervention (e.g., unexpected illness in the family).

On the PSI-SF Total, two parents demonstrated reliable change in Total parenting stress, two on Parental Distress, one on Parent–Child Dysfunctional Interaction and one on the Difficult Child subscales.

All four parents demonstrated reliable change on the CBCL Total, with three parents reporting reliable change on the CBCL Externalising subscale.

## Discussion

### Summary of Main Findings and Tentative Explanations

We conducted a preliminary evaluation of a parenting intervention, HHYP, which aims to improve the quality of the parent–child relationship, increase parent self-regulation and self-efficacy, and support young parents to respond more effectively to child behavioural and emotional problems. The study used an AB design plus follow-up, replicated across four dyads to refine the intervention and investigate treatment progress.

Overall, results suggest that several aspects of parent–child relationship quality improved over the course of the intervention. Linear mixed models using observational data revealed evidence of an improvement in reciprocity and parental sensitivity over the treatment phase, and no evidence for changes in child engagement, negative states, intrusiveness or withdrawal in the same phase, and no evidence of shifts in scores at the beginning or end of treatment. Single case parent-report demonstrated improvements in child behaviour and emotional problems, stress, parent self-regulation and parent self-efficacy and three parents reported improvements in depressive symptoms from baseline to follow-up. One parent demonstrated positive change in anxiety and two parents demonstrated improvements on parent stress.

While the dyads shared commonalities, including parent and toddler ages, they were relatively heterogeneous, with different trauma history, relationship support, ethnic backgrounds and child concerns. For example, Dyad 1 and 4 demonstrated greater motivation and openness to learn new skills and were in more stable living conditions than Dyad 2 and 3. These two dyads had less support overall, ongoing child protection involvement, more recent history of domestic violence and drug use and were in stressful, unstable intimate relationships and fearful of child removal. It is possible that these were contributing factors to the differing patterns of results between cases, as demonstrated by the individual visual graphs and RCIs. It would be beneficial to investigate if motivation, trauma history, current child protection involvement and mothers’ relationship stability are moderators of outcomes in future program evaluations.

### Integration of Results with Previous Findings

Findings are encouraging considering parent sensitivity and reciprocity are both integral for secure attachment and promoting healthy child socio-emotional development [22–25, 28]. Results from this study are consistent with prior intervention studies [71, 72], demonstrating that reciprocity and sensitivity can be improved with intervention. Both attachment- and social learning-based interventions focus on changing parenting behaviour in order to create change in the parent–child relationship and in child behaviour [e.g., 38–40, 73, 74]. However, even when interventions focus on changing parenting behaviours to generate improvement in parent–child relationships, few studies in the extant literature have measured session-by-session observational data of the process of relational change between young parents and their toddlers throughout interventions, which is a strength of this study.

Given improvements in the observed parent child-functioning, parent self-report findings may be indicative of relatively strong parental attributions about parent–child relationships, attributions that may have been derived from their own experience of early adversity. It may also reflect broad, more generalized concerns about their current situation and future potential. Improving maternal self-regulation, parent self-efficacy, depressive symptoms and child behaviour and emotional problems may have longer term benefits for these families. For instance, research has shown that maternal self-regulation difficulties moderate the relationship between maternal history of maltreatment and child behaviour and emotional problems [6] and increase the risk of maltreatment continuity [75]. Additionally, parent self-efficacy predicts or contributes to beneficial outcomes for the parent–child relationship, parenting competence and child socio-emotional development, and is negatively related to parental depression and anxiety [76–78]. The negative relationship between maternal depression and child wellbeing is well established, with findings showing that maternal depression is associated with parent irritability, hostility, intrusiveness, lower sensitivity and withdrawal [79, 80] and also related to higher levels of child problems, including internalising, externalising and general psychopathology [81]. Thus, improvements in these areas are promising.

While the mixed model did not demonstrate improvements in child engagement, individual analysis indicated that two of the four children demonstrated reliable change for engagement. Increases in parent sensitivity and child engagement are consistent with the biobehavioural synchrony model [32, 33] which proposes that parent and child interactions shape and reciprocally reorganise each other’s behaviour from moment to moment. There was a larger effect in our findings for parent sensitivity than child engagement, which may indicate the potential benefit of targeting parent sensitivity, as it is likely necessary to improve child engagement over time. It is possible that larger increases in child engagement take longer to develop in a population characterised by significant complexity and history of trauma. It is also possible that small magnitude changes in these observable behaviours accumulated to result in larger changes in the parent self-report measures of mental health, parent-self-efficacy, parent regulation and child behaviour. It is also possible that the parent and child engagement with the therapist contributed to improvements in self-report symptoms. This speculation would be an interesting avenue for future research.

### Limitations and Strengths

We acknowledge several limitations of this study. While the SCED methodology was designed rigorously, this population is both hard to reach and difficult to retain in treatment, which limited the number of completed cases and the data in the follow-up phase and follow-up time frame, limiting conclusions about longer term effects. These characteristics underline the necessity of pursuing treatment-based research in this area. The intervention was designed to be flexible to meet individual needs, however, this meant that not all participants had the same number of sessions, so it is not possible to draw definite conclusions about whether the number of sessions affect outcomes. We believe this flexibility in delivery is vital to help meet individual and complex needs. Dyad 1 had substantially more tele-health sessions, however, extra sessions did not seem to improve outcomes, so perhaps these did not contribute to substantive change. We also did not utilize a formal measure of social validity, nonetheless we gathered extensive qualitative data from participants and their support team that was used to refine the intervention. The qualitative feedback indicated that the HHYP intervention was socially relevant, acceptable and effective. Given the large amount of qualitative data collected over the course of the intervention, it will be reported in a separate publication. Further, this study was replicated across participants but not across settings or therapists and was, in addition, delivered by the intervention developers. Replication is, therefore, essential.

One of the strengths of this study was that it included repeated observational measures as well as parent-report. The combination of delivery methods, including in vivo coaching, video feedback and separate parent and dyad sessions that were guided by the treatment manual but also able to be tailored to individual needs is a strength of the HHYP intervention. As noted, there were potentially several moderating factors that may account for the differing pattern of results for each case. Factors such as current child protection involvement and mothers’ relationship stability appeared to impact their engagement, openness about parenting difficulties and development of trust with the therapist. Thus, we believe the capacity to develop strong therapeutic relationships, pace the program and target particular needs was integral to its effectiveness.

## Conclusions and Future Research

This study provides important insight into the process of relational change between young parents and their toddlers throughout a parenting intervention. It also provides preliminary support for the Holding Hands Young Parents intervention, delivered within the context of a comprehensive multifaceted service. Ongoing support from participants’ case managers was an important factor that facilitated engagement with our intervention. The feedback gained throughout, from both participants and staff will be used to refine the treatment manual.

While a randomized clinical trial (RCT) is often considered the “gold standard” to evaluate treatment efficacy, this is not an ethical option for this vulnerable population. Future research could extend our findings by including concurrent baselines with different starting times for each dyad. This would allow a comparison across series, which would strengthen causal inferences [82, 83]. We propose that the next research stage would be a larger cohort study replicated across settings and therapists, with formal measures of social validity and examination of potential moderators. Our findings demonstrate that it is possible to strengthen parent–child relationships for families who have experienced complex trauma and that young parents are willing and able to create positive changes to break intergenerational cycles of adversity if offered sufficient support.

## Summary

This study provides a preliminary evaluation of a dyadic intervention for young parents with history of complex trauma and their toddlers, *Holding Hands Young Parents* (HHYP). Four mothers (17–22 years) and toddlers (12–33 months) completed the intervention, which aims to improve parent–child relationships, parent self-regulation, parent self-efficacy and mental health, and child behaviour and emotional problems. We used an A–B single case experimental design series with follow-up and randomised baseline. HHYP is based on attachment theory, the biobehavioral synchrony model and social learning theory. After screening, dyads were randomised to two or three-week baseline control conditions and subsequently treated in a university-based psychology clinic. Baseline assessments included semi-structured clinical interviews, standardised parent-report measures and observational measures of parent–child relationship interactions. Biweekly observational assessments of the parent–child relationship and weekly parent-report measures occurred throughout. Linear mixed models revealed evidence of an improvement in reciprocity and parental sensitivity over the treatment phase, and no evidence for changes in child engagement, negative states, intrusiveness or withdrawal in the same phase, and no evidence of shifts in scores at the beginning or end of treatment. Reliable Change Index indicated improvement in parent-reported self-regulation, self-efficacy, stress and child emotional and behavioural problems from baseline to follow-up. Self-reported depression also showed significant reliable change for three of the four mothers. This study provides insight into the process of relational change between young parents and their toddlers over the course of HHYP and preliminary data on the HHYP protocol. Further replication and evaluation are needed.

### Supplementary Information

Below is the link to the electronic supplementary material.

Supplementary file1 (DOCX 14 KB)

## Footnotes

1 One dyad discontinued because the child was removed from the mother’s care by child protection services, two others due to extreme family stress. The fourth dyad moved from her supported residential home to a location that prohibited her travelling to the university clinic.

2 The formula is: RCI = (*x*^1^ – *x*.^2^) / S_*E*_*,* where S_*E*_ = *SD √*1 – *r (“r” refers to internal consistency: Cronbach’s alpha coefficients were used).*
