# Supplementary material for: Improving Parent–Child Relationships for Young Parents in the Shadow of Complex Trauma: A Single-Case Experimental Design Series
Source: Child Psychiatry Hum Dev. 2022 Jun 27;55(1):94–106. doi: 10.1007/s10578-022-01379-8 (PMC10796421; doi:10.1007/s10578-022-01379-8)
Supplement: Supplementary file 1 — Supplementary file1 (DOCX 14 KB) [file 10578_2022_1379_MOESM1_ESM.docx]

Table 1 Supplementary

*Number of sessions completed for each dyad per phase*

| Participant | Baseline | Intervention | Follow-up |
| --- | --- | --- | --- |
| Dyad 1 | 6 | 21 | 5 |
| Dyad 2 | 6 | 14 | 2 |
| Dyad 3 | 4 | 13 | 3 |
| Dyad 4 | 4 | 14 | 4 |
